# Prognostic Significance and Functional Role of PPIB in a Retrospective Cohort of Patients with Advanced Gastric Cancer

**DOI:** 10.32604/or.2026.083437

**Published:** 2026-08-13

**Authors:** Kyungeun Kim, Eunho Cho, Eun Joo Chung, Kwon-Ho Song, Tae Woo Kim, Seoung Wan Chae, Joon-Yong Chung

**Affiliations:** 1 Department of Pathology, Kangbuk Samsung Hospital, Sungkyunkwan University School of Medicine, Seoul, Republic of Korea; 2 Pathology Center, Seegene Medical Foundation, Seoul, Republic of Korea; 3 Department of Convergence Medicine, College of Medicine, Korea University, Seoul, Republic of Korea; 4 Radiation Oncology Branch, Center for Cancer Research, National Cancer Institute, National Institutes of Health, Bethesda, MD, USA; 5 Department of Cell Biology, Daegu Catholic University School of Medicine, Daegu, Republic of Korea; 6 BK21 Graduate Program, Department of Biomedical Science, Korea University College of Medicine, Seoul, Republic of Korea; 7 Center for Cancer Research, National Cancer Institute, National Institutes of Health, Bethesda, MD, USA

**Keywords:** Gastric cancer, peptidyl-prolyl isomerase B, cyclophilin B, prognostic biomarker, tumorigenicity

## Abstract

**Objectives:** Gastric cancer remains a major global health burden, and robust biomarkers are needed to improve risk stratification. Although peptidyl-prolyl isomerase B (PPIB) has been suggested as a potential oncogenic factor, its clinical utility in gastric cancer remains unclear. This study aims to evaluate the clinicopathological and prognostic significance of *PPIB* mRNA expression and delineate its functional role in driving tumor progression. **Methods:** *PPIB* mRNA expression was evaluated in a retrospective cohort of 497 gastric cancer patients using RNAscope *in situ* hybridization and digital image analysis. Functional roles and underlying mechanism were assessed through siRNA-mediated knockdown, plasmid-driven overexpression, and STAT3-targeted rescue experiments *in vitro*. **Results:** High *PPIB* expression was significantly associated with aggressive clinicopathological features, including larger tumor size, advanced T stage, and lymph node metastasis. Patients with high *PPIB* expression had significantly worse progression-free survival (PFS) and overall survival (OS) compared to those with low expression, particularly in advanced stage. Multivariate analysis identified high *PPIB* expression as an independent predictor of poor PFS and OS. Functionally, *PPIB* silencing suppressed gastric cancer cell proliferation, migration, and invasion, whereas its overexpression enhanced these oncogenic behaviors in both cancerous and non-tumorigenic cells. These aggressive phenotypes were effectively mitigated through STAT3 targeted functional rescue experiments. **Conclusions:** PPIB is a robust independent prognostic biomarker for gastric cancer, especially in advanced stages. Its role in driving aggressive phenotypes via STAT3 signaling underscore its potential for risk stratification and as a therapeutic target.

## 1 Introduction

Gastric cancer continues to represent a substantial global disease burden, ranking as the fifth most commonly diagnosed malignancy and the third leading cause of cancer-related mortality worldwide [1]. It is also among the most common malignancies affecting both men and women in South Korea [2]. While early gastric cancer (EGC) is often effectively managed via endoscopic resection, with the 5-year disease-specific survival rate of patients typically exceeding 90%, advanced gastric cancer (AGC) has a 5-year survival rate that often falling below 10% [3,4]. Despite recent therapeutic advancements, clinical outcomes remain highly variable even among patients with similar clinicopathologic features. This disparity is particularly evident in advanced stages, where conventional staging systems often provide limited prognostic resolution. Consequently, there is an urgent unmet need for molecular tools that enable more precise, individualized patient management [5,6].

Recent studies have highlighted the pivotal contribution of molecular chaperone systems to tumor biology. Beyond their canonical roles in protein folding, these proteins actively modulate intracellular signaling networks that support malignant growth, stress adaptation, and therapeutic resistance [7,8]. In this context, peptidyl-prolyl isomerase B (PPIB), also known as cyclophilin B (CypB), has emerged as a critical regulator of protein conformation. It is an endoplasmic reticulum–resident member of the cyclophilin family encoded by the *PPIB* gene [9]. As a cis-trans isomerase, CypB facilitates protein folding and serves as an intracellular receptor for cyclosporine A [10,11]. Beyond its physiological roles in immunoregulation and bone homeostasis [12,13,14], accumulating evidence implicates CypB in cancer progression. Elevated expression of CypB has been reported in multiple malignancies including breast, brain, liver, colon, and lung cancers [15,16,17,18,19,20,21]. In gastric cancer, a prior study demonstrated that CypB is highly expressed in gastric cancer tissues, and that downregulation of PPIB suppresses cancer cell growth and proliferation [22]. Furthermore, elevated levels of CypB in both patient sera and tumor tissues correlate with advanced tumor stage and reduced overall survival [23]. However, these findings have largely been limited to small-scale cohorts or basic functional assays. Consequently, the specific prognostic value and clinical utility of *PPIB*, particularly in advanced-stage disease remain to be fully elucidated.

The purpose of this study was to evaluate the clinicopathological and prognostic significance of *PPIB* mRNA expression in a large, well-characterized cohort encompassing the full clinical spectrum of the disease. We hypothesized that PPIB acts as a key driver of tumor aggressiveness and serves as an independent prognostic determinant, particularly for risk stratification in advanced gastric cancer. To test this, we integrated high-resolution *in situ* hybridization (ISH) analysis with functional and mechanistic validation in gastric cell lines, thereby demonstrating that PPIB promotes oncogenic phenotypes such as proliferation and invasion through a downstream pathway that is functionally dependent on STAT3 activation.

## 2 Materials and Methods

### 2.1 Study Cohort and Tissue Specimens

This study was conducted in accordance with the ethical principles outlined in the Declaration of Helsinki and received approval from the Institutional Review Board of Kangbuk Samsung Hospital (approval no. KBSMC IRB 2025-11-043). The requirement for informed consent was waived by the IRB due to the retrospective nature of this study. We retrospectively reviewed 529 consecutive patients who underwent gastrectomy or endoscopic resection for primary gastric cancer at Kangbuk Samsung Hospital (Seoul, South Korea) between January 2011 and December 2014. The patient selection process, including specific exclusion details, is summarized in the CONSORT flow diagram (Supplementary Fig. S1). Patients presenting with recurrent or metastatic disease at the time of treatment or lacking available formalin-fixed, paraffin-embedded (FFPE) tissue blocks were excluded (n = 32). The final study cohort comprised 497 patients. Clinical and pathological data, including age, sex, and follow-up outcomes, were collected from electronic medical records. Clinical outcomes were monitored from the date of primary surgical resection or endoscopic procedure, which served as the time origin for all survival analyses. Progression-free survival (PFS) was defined as the interval from the date of surgery to the date of first documented disease progression (locoregional recurrence or distant metastasis) or death from any cause. Overall survival (OS) was defined as the interval from the date of surgery to the date of death due to gastric cancer. Patients who were alive and event-free at the time of the last clinical follow-up were censored for both PFS and OS analyses. Given that OS strictly reflects cancer-specific mortality, any late-stage non-cancer competing risks mathematically account for discrete variations between the two endpoints without violating statistical consistency. The clinicopathological profiles of the patients included in this study are detailed in Supplementary Table S1.

### 2.2 Pathologic Diagnosis and Tissue Microarray Generation

Gastrectomy specimens were fixed in 10% neutral-buffered formalin at room temperature for 24 h immediately following surgical resection and embedded in paraffin using standard procedures. Hematoxylin and eosin (H&E) slides were independently reviewed by pathologists (KK and SWC), who were blinded to clinical outcomes and other clinicopathological information during the review, to determine histological subtypes according to the World Health Organization (WHO) and Lauren classifications. Any discrepancies between the two pathologists were resolved by consensus review using a multi-head microscope. Pathologic staging, including tumor depth (pT) and lymph node involvement (pN), was documented, along with the presence of lymphovascular invasion (LVI), perineural invasion (PNI), and peritumoral dysplasia. For tissue microarray (TMA) construction, representative tumor regions were identified on the donor blocks. A single 2 mm core was extracted from each case and incorporated into the recipient TMA block. Tonsil tissue was incorporated into the TMA as a control core, and whole-tissue sections containing normal gastric mucosa were utilized as external controls to ensure staining quality control.

### 2.3 EBV In Situ Hybridization

To facilitate molecular subtyping of gastric cancer, ancillary assays were performed to assess molecular status, including mismatch repair (MMR) protein expression and Epstein-Barr virus (EBV) infection. EBV status was evaluated by EBV-encoded RNA ISH (EBER-ISH) using the BOND Ready-to Use EBER probe (Leica Biosystems, PB0589, Vista, CA, USA), which is a fluorescein-labeled oligonucleotide probe. The hybridization signals were visualized using the BOND polymer refine detection kit (Leica Biosystems, DS9800) on an automated platform (Leica BOND-III Automated IHC/ISH Stainer; Leica Biosystems). The EBER-ISH assay was performed on whole-tissue sections. Cases were classified as EBV-positive when tumor cells exhibited diffuse and strong nuclear localization of the hybridization signals.

### 2.4 Microsatellite Instability Analysis

Microsatellite instability (MSI) status was determined using the U-TOP MSI Detection Kit (Seasun Biomaterials, IVD17-642, Daejeon, South Korea) by analyzing five mononucleotide markers (NR21, NR24, NR27, BAT25, and BAT26) as previously established [24]. Briefly, genomic DNA was extracted from FFPE tissue sections using the QIAamp DNA FFPE Tissue Kit (Qiagen, #56404, Germantown, MD, USA) according to the manufacturer’s protocol. DNA concentration and purity were assessed using a NanoDrop One spectrophotometer (Thermo Fisher Scientific, Waltham, MA, USA), and only samples with an A_260_/A_280_ ratio ≥ 1.6 and a minimum concentration of 10 ng/μL were accepted for downstream analysis. For the multiplex real-time PCR, a total reaction volume of 20 μL (comprising 5 μL of extracted DNA and 15 μL of master mix) was amplified using an ABI 7500 Real-Time PCR System (Applied Biosystems, Foster City, CA, USA). The cycling conditions consisted of an initial denaturation at 95°C for 5 min, followed by 35 cycles of 95°C for 30 s, 58°C for 30 s, and 72°C for 30 s, employing peptide nucleic acid (PNA) probe-based melting curve analysis to detect sequence deletions through Tm shifts. Based on the standardized National Cancer Institute guideline [25], tumors were initially screened for instability in the five markers. For clinical relevance, MSI status was categorized as either MSI-high (MSI-H), which was characterized by instability in ≥2 markers, or microsatellite stable (MSS), which exhibited instability in fewer than two markers.

### 2.5 RNAscope In Situ Hybridization

Detection of mRNA transcripts was performed on 5-μm FFPE gastric cancer TMA sections using the RNAscope 2.5 HD Reagent Kit–Brown (Advanced Cell Diagnostics [ACD], #322310, Newark, CA, USA) according to the manufacturer’s instructions. Briefly, tissue sections were deparaffinized and subjected to heat-induced target retrieval in RNAscope Target Retrieval Reagent (ACD, 322000) at 95–98°C for 15 min, followed by protease digestion with RNAscope Protease Plus (ACD, 322331) at 40°C for 30 min. Probe hybridization was carried out for 2 h at 40°C in a HybEZ™ hybridization oven (ACD). The Hs-PPIB probe (ACD, #313901) was used to detect *PPIB* mRNA expression, while the bacterial gene dapB probe (ACD, #310043) served as a negative control. Following sequential signal amplification steps, chromogenic detection was performed using 3,3′-diaminobenzidine (DAB), which is provided as a component of the aforementioned detection kit (ACD, #322310), producing brown punctate signals corresponding to individual RNA transcripts. Sections were counterstained with hematoxylin, dehydrated through graded ethanol and xylene, and permanently mounted. TMA-slide images were acquired using an Aperio AT2 scanner (Leica Biosystems) at 40× magnification for subsequent histopathological evaluation and quantification. Transcript abundance was evaluated via digital image analysis. For the quantification, tumor regions within the TMA cores were selectively identified and annotated by the pathologists, while stromal, necrotic, inflammatory, or artifact-containing areas were strictly excluded from the analysis. Detailed descriptions of the specific quantification parameters, the total number of analyzed fields/cores, and the predefined scoring criteria used to classify cases into low versus high expression are detailed in the subsequent section.

### 2.6 QuPath Analysis of PPIB RNAscope Image

Tumor regions were manually annotated by a board-certified pathologist (KK) to ensure histological accuracy. To minimize observer-dependent bias, the donor tumor areas for TMA construction had been independently reviewed and validated by two pathologists, whereas subsequent digital annotations were performed by a single investigator using standardized criteria to maintain intra-analysis consistency. Digital image analysis for the high-resolution quantification of *PPIB* mRNA expression was performed using QuPath (version 0.5.1) [26]. To assess analytical reproducibility, the workflow was validated by repeated analyses on a randomly selected subset of cores (10% of the total), and intra-operator reproducibility was evaluated using the intraclass correlation coefficient (ICC) analysis. Following the importation of TMA-slide images, the image type was designated as ‘BRIGHTFIELD_OTHER’ and color deconvolution was executed by applying calibrated stain vectors for Hematoxylin and DAB. Individual cells were then identified through the StarDist extension [27], with core segmentation parameters precisely set as follows: pixel size of 0.5 μm, cell expansion of 5.0 μm, and a cell constraint scale of 1.5 to delineate precise cytoplasmic boundaries and ensure comprehensive signal inclusion. To achieve cell-type-specific quantification, a machine learning-based Random Trees classifier built within QuPath was trained to categorize individual cells into tumor, stromal, and immune classes. The classifier was trained using over 200 manually annotated cell examples across multiple cores, extracting features including cell morphology, nucleus/cytoplasm intensity, and texture metrics. A k-fold cross-validation strategy was applied, achieving an overall classification accuracy of > 92% during training. This classification process was rigorously verified by a board-certified pathologist to ensure that PPIB signals were accurately assigned to their respective cellular compartments, minimizing potential interference from the surrounding stroma or inflammatory cells. Within these classified cell boundaries, subcellular detection was subsequently employed to quantify *PPIB* mRNA transcripts, where signals were categorized as individual spots measuring 0.3–2 μm^2^ or clusters exceeding 2 μm^2^. Final expression levels were calculated as the estimated number of spots and clusters per unit area (mm^2^) within the annotated tumor regions, providing a normalized quantitative metric of PPIB transcript density for each case.

### 2.7 Cells, Cell Culture and Generation of Cell Lines

Human gastric cancer cells were purchased from Korean Cell Line Bank (KCLB, Seoul, South Korea), including SNU719 (KCLB, #00719), MKN28 (KCLB, #80102), AGS (KCLB, #21739), and SNU601 (KCLB, #00601). The human gastric cancer cell line YCC2 was obtained from the Yonsei Cancer Center (YCC, #KC0277, Seoul, South Korea), and the human bronchial epithelial (HBE) cell line IB3-1/C38 (C38) was purchased from the American Type Culture Collection (ATCC, JHU-54, Manassas, VA, USA). SNU719, MKN28, SNU601, YCC2, and C38 cells were maintained in RPMI 1640 medium (Welgene, LB001-01, Gyeongsan, Gyeongsangbuk do, South Korea), whereas AGS cells were cultured in Dulbecco’s Modified Eagle’s Medium (DMEM) (Welgene, LM001-05). All culture media were supplemented with 10% fetal bovine serum (FBS) (Welgene, S101-01) and 1% antibiotics (Welgene, S203-01). All cell lines were tested for mycoplasma using the Mycoplasma Detection Kit (Thermo Fisher Scientific, #4460623) and grown at 37°C in a 5% CO_2_ incubator/humidified chamber. The authenticity of all cell lines was verified by short tandem repeat (STR) profiling performed by IDEXX Laboratories, Inc. (Westbrook, ME, USA), and the cells were utilized within 6 months of testing.

For the generation of stable PPIB overexpressing cell lines, designated as C38-PPIB and SNU719-PPIB, C38 and SNU719 cells were seeded in 90 mm cell culture dishes and grown to approximately 70% confluence. Cells were then transfected with 5 μg of pCMV3-PPIB-FLAG plasmid (Sino Biological, HG11124-CF, Beijing, China) or an empty vector control using Lipofectamine 2000 (Thermo Fisher Scientific, #11668027) according to the manufacturer’s instructions. At 48 h post transfection, the transfection medium was replaced with fresh complete medium. To select stable transformants, cells were treated with 300 μg/mL of Hygromycin B (Duchefa Biochemie, H0192, Haarlem, Netherlands) for 14 days, with the selection medium refreshed every 2 to 3 days. Successful establishment of stable cell lines was confirmed when non transfected control cells under the same selection conditions reached complete cell death, while the hygromycin resistant cells survived and continuously proliferated. Hygromycin resistant pooled cell populations were then expanded and maintained in complete medium containing 150 μg/mL of Hygromycin B for subsequent experiments. Stable overexpression of PPIB in the pooled populations was validated via Western blotting using an anti PPIB antibody.

### 2.8 siRNA Constructs

Synthetic small interfering RNAs (siRNAs) specific for GFP and PPIB were purchased from Bioneer (Daejeon, Korea): Non-specific GFP (green fluorescent protein), 5′-GCAUCAAGGUGAACUUCAA-3′ (sense), 5′-UUGAAGUUCACCUUGAUGC-3′ (antisense); PPIB-#1, 5′-CAGCAAAUUCCAUCGUGUA-3′ (sense), 5′-UACACGAUGGAAUUUGCUG-3′ (antisense); PPIB-#2, 5′-CUUAGCUACAGGAGAGAAA-3′ (sense), 5′-UUUCUCUCCUGUAGCUAAG-3′ (antisense); PPIB-#3, 5′-GUGUAUUUUGACCUACGAA-3′ (sense), 5′-UUCGUAGGUCAAAAUACAC-3′ (antisense), and STAT3, 5′-CAGCAAAAAGUUUCCUACA-3′ (sense), 5′-UGUAGGAAACUUUUUGCUG-3′ (antisense). For transient knockdown experiments, cells were seeded in 6-well culture plates at a density of 2 × 10^5^ cells per well. Upon reaching approximately 60% to 70% confluence, cells were transfected with 100 pmol of synthesized siRNAs using Lipofectamine 2000 (Thermo Fisher Scientific, #11668027) at a 1:2 ratio of siRNA to transfection reagent (100 pmol siRNA to 2 μL Lipofectamine 2000). Transfections were performed in serum free and antibiotic free Opti MEM reduced serum medium (Thermo Fisher Scientific, #31985062). After a transfection duration of 6 h, the transfection medium was replaced with fresh complete growth medium appropriate for each cell line. To evaluate the knockdown efficiency of PPIB and STAT3, cells were harvested at specific time points post transfection. Validation of silencing efficiencies was performed at both the mRNA level via quantitative reverse transcription polymerase chain reaction at 24 h post transfection, and at the protein level via Western blotting at 48 h post transfection. All subsequent downstream assays were systematically performed at 48 h after transfection.

### 2.9 Western Blot Analysis

Lysate extracted from a total of 5 × 10^5^ cells was used to perform Western blot analysis as described previously [28]. Briefly, cells were lysed in RIPA buffer (Elpis Biotech, EBA-1149, Daejeon, South Korea), supplemented with 10 μM leupeptin (Thermo Fisher Scientific, #78435), 2 μg/mL aprotinin (Thermo Fisher Scientific, #78432), and 50 mM NaF (Thermo Fisher Scientific, #424320050). Lysates were incubated on ice for 30 min and centrifuged at 13,000× *g* for 20 min at 4°C. Protein concentrations were determined using a BCA protein assay (Thermo Fisher Scientific, #23225). Equal amounts of protein (15 μg per lane) were separated on 12% SDS-PAGE gels and subsequently transferred to PVDF membranes (Thermo Fisher Scientific, LC2002) at 90 V for 90 min at 4°C. The membranes were blocked with 5% skim milk for 1 h at room temperature. They were then incubated overnight at 4°C with the following primary antibodies diluted at a 1:3000 ratio: anti-PPIB (Sino Biological, 101242-T36), anti-Phospho-STAT3 (pSTAT3, Cell Signaling Technology, #9145, Danvers, MA, USA), anti-STAT3 (Cell Signaling Technology, #12640), and anti β-Actin (MBL Life Science, M177-3, Tokyo, Japan) as an internal loading control. After washing with TBS-T, the membranes were incubated with horseradish peroxidase-conjugated secondary antibodies, goat anti-rabbit IgG (ADI-SAB-300-J) or goat anti-mouse IgG (ADI-SAB-100-J), both from Enzo Life Sciences, Inc. (Farmingdale, NY, USA), at a dilution of 1:5000 for 2 h at room temperature. Following another wash with TBS-T, immune-reactive bands were developed using a chemiluminescence ECL detection system (Elpis Biotech, EBP-1073). Protein signals were detected and captured using a luminescent image analyzer (LAS-4000 Mini, Fujifilm, Tokyo, Japan). The intensity of the Western blot signals was quantified using Multi Gauge software (version 2.0; Fujifilm). All Western blot analyses were performed using three independent biological replicates (n = 3), where cells were independently seeded, cultured, treated, and processed for fresh protein lysates. Representative blot images are displayed in the figures. Where applicable, densitometric quantification was performed across the three independent biological replicates and is presented as the mean ± standard deviation (SD).

### 2.10 Trypan Blue Exclusion Assay

To evaluate cell proliferation, viable cell numbers were determined using the Trypan blue exclusion method. Briefly, AGS, MKN28, SNU719, and C38 cells were seeded at a density of 3 × 10^3^ cells/well in 48-well plates and cultured under the indicated experimental conditions for up to 72 h. Proliferation assays were performed every 24 h (at 24, 48, and 72 h after seeding). At each specified time point, both floating and adherent cells were harvested, with the adherent population detached using trypsin-EDTA. The resulting cell suspension was mixed with 0.4% Trypan blue solution (Thermo Fisher Scientific, #15250061) at a 1:1 ratio and incubated for 2 min at room temperature. To prevent the overestimation of non-viable cells, stained cells were counted immediately using a hemocytometer under an inverted light microscope (Model No. CKX53; Olympus Corporation, Tokyo, Japan). All experiments were conducted using three independent biological replicates, with each biological replicate comprising three technical replicate wells per condition at each time point.

### 2.11 Colony Formation Assay

Cells were seeded at a density of 500 cells/well in 12-well cell culture plates. The cells were then incubated at 37°C for 7–14 days, depending on the specific cell line, until clearly visible colonies of ≥1 mm in diameter had formed. After the incubation period, the culture medium was removed, and the colonies were simultaneously fixed and stained for 10 min at room temperature using 0.5% crystal violet prepared in methanol (Sigma-Aldrich, C0775, St. Louis, MO). Excess stain was thoroughly removed by washing the plates with de-ionized water. Stained colonies of diameter 1 mm were counted manually from microscopic images (Model No. CKX53; Olympus Corporation, Tokyo, Japan). Each colony formation assay was carried out in triplicate and repeated three times.

### 2.12 Wound-Healing Assay

Cell migration capacity was determined by scratch wound-healing assay. Cells were seeded at a density of 8 × 10^5^ cells/well in 6-well cell culture plates and incubated until they reached a 100% confluent monolayer state. A linear scratch wound was generated through the center of each well using a sterile 1000-μL pipette tip held perpendicular to the plate surface. To ensure consistency among samples, all scratches were generated by a single operator, and any wells displaying irregular scratches or detached monolayers were excluded from the analysis. After scratching, free-floating and detached cells were removed by washing with phosphate-buffered saline. The medium was then replaced with fresh, low-serum medium containing 0.1% FBS to minimize the confounding effects of cell proliferation on wound closure. For AGS cells, low-serum DMEM (Welgene, LM001-05) supplemented with 0.1% FBS (Welgene, S101-01) and 1% antibiotics (Welgene, S203-01) was utilized. For MKN28, SNU719, and C38 cells, low-serum RPMI medium (Welgene, LB001-01) supplemented with 0.1% FBS (Welgene, S101-01) and 1% antibiotics (Welgene, S203-01) was used. To ensure consistency between time points, the underside of each plate was marked prior to imaging. Images of the same wound regions within each well were captured at 0 h and 16 h post-scratching using an Olympus CKX53 inverted microscope (Olympus Corporation) equipped with a 4× objective lens. Cell migration was quantified by measuring the wound width from three fields analyzed per well using ImageView software (version 4.11; BestScope International Limited, Beijing, China). Wound closure was calculated as the percentage decrease in wound width at 16 h relative to the initial wound width at 0 h. Each assay was performed in three independent biological replicates, with each biological replicate encompassing three technical replicate wells per experimental condition. Quantitative data were summarized as the mean ± SD from three independent experiments, and statistical significance was determined using the unpaired two-tailed Student’s *t*-test.

### 2.13 In Vitro Invasion Assay

The invasion ability of tumor cells was examined using 24-well culture insert-based assays (BD Biosciences, #353097, Franklin Lakes, NJ). The culture insert, with an 8 μm pore size, was precoated to a density of 2–3 mg/mL insert of 20 μL Matrigel Basement Membrane Matrix (BD Biosciences, CLS354230) at a density of 2–3 mg/mL in a volume of 20 μL per insert. Cells were suspended in serum free medium, and 1 × 10^5^ cells were added onto the insert. To serve as a chemoattractant, the lower chamber was filled with complete culture medium containing 10% FBS (Welgene, S101-01). For AGS cells, DMEM (Welgene, LM001-05) was used, whereas RPMI medium (Welgene, LB001-01) was utilized for MKN28, SNU719, and C38 cells; no additional chemoattractants were supplemented. After incubating for 24 h at 37°C, the cells that invaded or migrated through the Fluoro-Blok membrane were fixed and stained with 0.5% crystal violet prepared in methanol (Sigma-Aldrich, C0775). Images of the stained, invasive cells were acquired from each well using an Olympus CKX53 inverted microscope (Olympus Corporation) equipped with a 10× objective lens, and the number of invasive cells was subsequently counted. The samples were plated in triplicate, and the experiment was repeated at least three times.

### 2.14 Statistical and Bioinformatics Analysis

Statistical analyses were performed using the SPSS Statistics for Windows, version 26 (IBM Corp., Armonk, NY, USA). Associations between categorical variables were assessed using χ^2^ or Fisher’s exact tests. For the bioinformatics evaluating biomarker thresholding, the optimal cut-off for PPIB expression was determined using maximally selected rank statistics via the Web-R platform (http://web-r.org) and the maxstat R package. This approach accounts for non-linear relationships by evaluating all possible split points while adjusting *p*-values for multiple testing to minimize Type I error inflation. To ensure the stability and robustness of the identified threshold, internal validation was conducted using bootstrapping, and a sensitivity analysis was performed to compare the prognostic power. Survival curves for PFS and OS were analyzed using the Kaplan-Meier method and compared using the log-rank test. Cox proportional hazards regression models, including both univariate and multivariate approaches, were employed to identify independent prognostic factors. Variables demonstrating statistical significance (*p* < 0.05) in the univariate analysis were entered into the multivariate model using an enter method to calculate adjusted Hazard Ratios (HRs) and 95% confidence intervals (CIs). The incremental prognostic value of PPIB beyond the conventional Tumor Node Metastasis (TNM) staging system was assessed by comparing the performance of the TNM only model and the combined model (TNM+PPIB). The predictive accuracy was measured using Harrell’s concordance index (C-index) for OS and the time-dependent area under the receiver operating characteristic curve (AUC) for recurrence. The statistical significance of the improvement in model performance was validated using a bootstrapping method with 500 iterations. All statistical tests were two-sided, and *p* < 0.05 was considered statistically significant.

## 3 Results

### 3.1 High PPIB Expression Correlates with Aggressive Clinicopathologic Features in Gastric Cancer

A total of 497 patients with gastric cancer were included. The patients were treated by subtotal gastrectomy (n = 281, 56.5%), endoscopic submucosal resection (n = 130, 26.2%), total gastrectomy (n = 84, 16.9%), and proximal gastrectomy (n = 2, 0.4%). Lymph node dissection was performed in 362 patients. The mean age at diagnosis was comparable between the PPIB-high and PPIB-low groups (62.2 ± 12.6 vs. 60.9 ± 11.9 years). Over a mean follow-up of 54.5 months, 58 patients (11.7%) died of gastric cancer, while local recurrence and distant metastasis occurred in 27 (5.4%) and 38 (7.6%) patients, respectively.

Prior to clinicopathological correlation, the digital image analysis workflow was validated, demonstrating high analytical and intra-operator reproducibility with an ICC greater than 0.95 upon repeated subset analyses. Furthermore, the machine learning-based Random Trees classifier achieved an overall cell classification accuracy of >92% during training, ensuring reliable cell-type-specific quantification.

**Figure 1:**
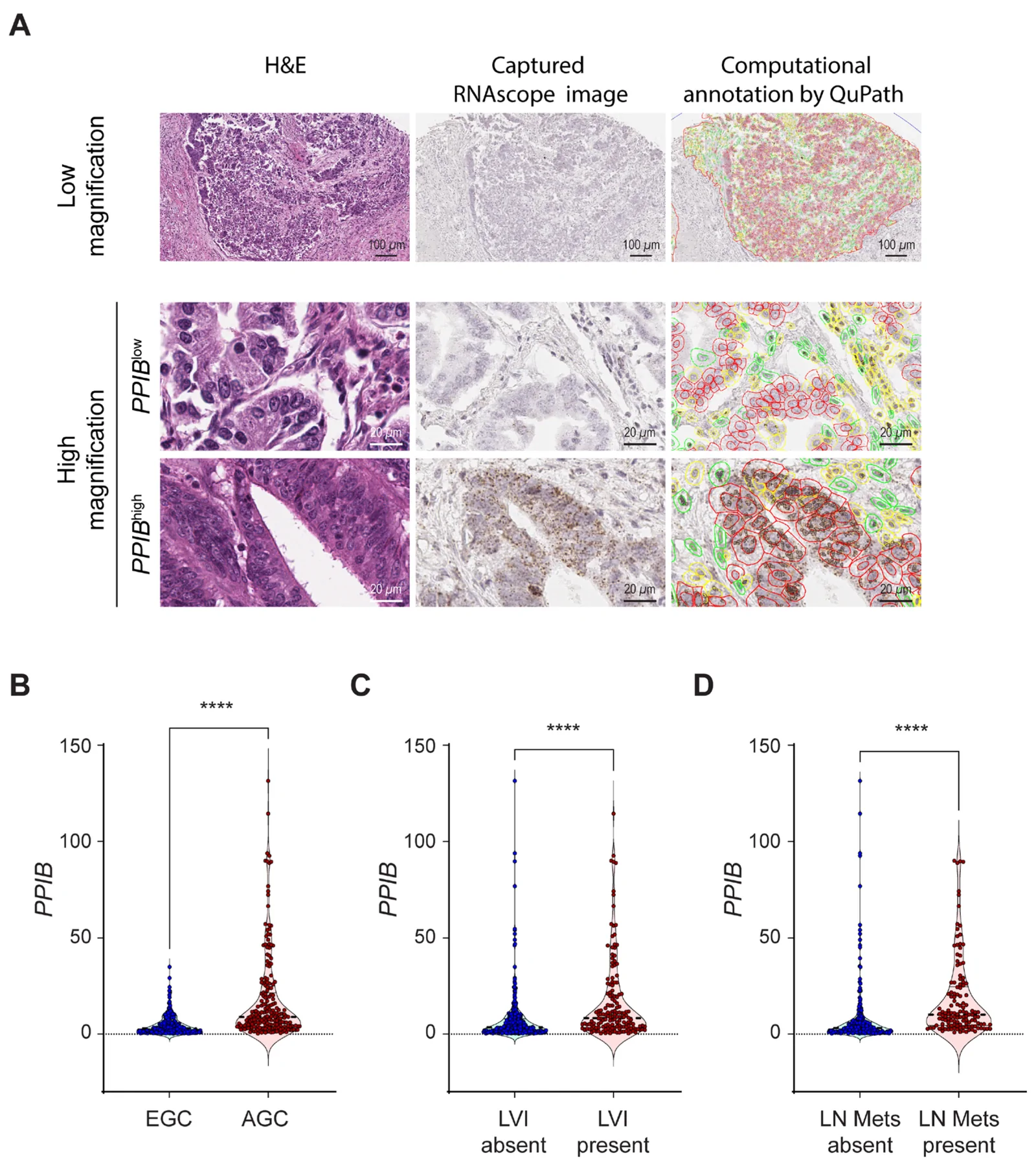
Spatial distribution and clinical relevance of *PPIB* expression in gastric cancer. (**A**) Representative images showing *PPIB* mRNA expression detected by RNAscope *in situ* hybridization (ISH) in gastric cancer tissues. Panels display H&E staining (left), RNAscope ISH signals (middle), and computational annotations (right). Images are shown at low magnification (top panel, scale bar, 100 μm) and under higher magnification (bottom panel, scale bar, 20 μm) for representative cases with *PPIB*^low^ and *PPIB*^high^ expression. Individual cells were computationally classified into cancer cells (red), stromal fibroblasts (green), and inflammatory cells (yellow). Brown punctate signals represent individual *PPIB* mRNA transcripts. (**B**–**D**) Relationship between *PPIB* mRNA expression and clinicopathological features of gastric cancer. *PPIB* expression was significantly higher in (**B**) advanced gastric cancer (AGC) vs. early gastric cancer (EGC), (**C**) tumors with lymphovascular invasion (LVI present) vs. absent, and (**D**) patients with lymph node metastasis (LN Mets present) vs. absent. Each point represents an individual patient sample. Statistical significance was assessed using the Mann-Whitney U test (****p < 0.0001).

RNAscope ISH analysis showed that *PPIB* expression was localized to discrete cytoplasmic puncta and clustered foci (Fig. 1A). Using the statistically validated optimal cut-off value of 2.74, which was derived via internal bootstrapping (95% confidence interval: 2.66–14.85; Supplementary Fig. S2), the study cohort was stratified into PPIB-high (n = 328, 66.0%) and PPIB-low (n = 169, 34.0%) groups. Sensitivity analysis justified the selection of this data-driven threshold over a simple median split (4.56), as the 2.74 threshold demonstrated vastly superior prognostic power, yielding a substantially higher Wald statistic (16.60 vs. 11.75) and a more pronounced HR (11.20 vs. 2.69) solely during this threshold evaluation. High *PPIB* expression was strongly associated with adverse tumor characteristics. Tumors in the PPIB-high group were significantly larger (4.5 ± 3.3 cm vs. 3.1 ± 2.0 cm; *p* < 0.001) and exhibited an advanced pathological stage more frequently. The prevalence of advanced T-stage disease (pT3–pT4) was markedly increased in the PPIB-high cohort (43.0% vs. 11.3%; *p* < 0.001), along with a substantially higher incidence of lymph node metastasis (47.5% vs. 11.9%; *p* < 0.001). Consistently, quantitative analyses confirmed higher *PPIB* expression in advanced versus early gastric cancer (Fig. 1B), as well as in tumors with LVI and nodal metastasis (Fig. 1C,D).

Histopathologically, elevated *PPIB* expression correlated with increased lymphatic (37.2% vs. 17.8%), vascular (11.9% vs. 0.6%), and perineural invasion (27.4% vs. 10.7%) (all *p* < 0.001). Clinically, the PPIB-high group demonstrated higher rates of distant metastasis (10.7% vs. 1.8%; *p* = 0.001) and local recurrence (8.2% vs. 0.0%; *p* < 0.001). However, *PPIB* expression was not associated with baseline demographic or histologic parameters, including age, sex, WHO classification, Lauren classification, or signet-ring cell component. No significant relationship was observed between *PPIB* expression and metabolic or nutritional status, as reflected by body mass index (BMI; *p* = 0.094; Table 1), with comparable distributions across BMI categories in both groups.

**Table 1:** The correlation between *PPIB* expression and clinicopathologic features of gastric cancer.

Characteristics	Low, n (%) *n* = 169	High, n (%) *n* = 328	*p* Value
**Age (years), mean ± SD**	60.9 ± 11.9	62.2 ± 12.6	0.256
**Tumor size (cm), mean ± SD**	3.1 ± 2.0	4.5 ± 3.3	<0.001
**Gender**			0.282
male	127 (75.1)	230 (70.1)	
female	42 (24.9)	98 (29.9)	
**BMI**			0.094
underweight (<18.5)	6 (3.6)	26 (7.9)	
normal (18.5–24.9)	101 (60.1)	209 (63.7)	
overweight (25.0–29.9)	55 (32.7)	80 (24.4)	
obesity (≥30)	6 (3.6)	13 (4.0)	
**Signet-ring cell component**			0.767
absent	114 (67.5)	227 (69.2)	
present	55 (32.5)	101 (30.8)	
**WHO classification**			0.496
tubular	101 (59.8)	188 (57.3)	
poorly cohesive	41 (24.3)	80 (24.4)	
mucinous	0 (0.0)	5 (1.5)	
carcinoma with lymphoid stroma	7 (4.1)	10 (3.1)	
mixed	20 (11.8)	45 (13.7)	
**Lauren classification**			0.711
intestinal	100 (59.2)	188 (57.3)	
diffuse	45 (26.6)	84 (25.6)	
mixed	24 (14.2)	56 (17.1)	
**Resection margin**			0.095
absent	169 (100.0)	320 (97.6)	
present	0 (0.0)	8 (2.4)	
**Lymphatic invasion**			<0.001
absent	139 (82.2)	206 (62.8)	
present	30 (17.8)	122 (37.2)	
**Vascular invasion**			<0.001
absent	168 (99.4)	289 (88.1)	
present	1 (0.6)	39 (11.9)	
**Lymphovascular invasion**			<0.001
absent	139 (82.2)	198 (60.4)	
present	30 (17.8)	130 (39.6)	
**Perineural invasion**			<0.001
absent	151 (89.3)	238 (72.6)	
present	18 (10.7)	90 (27.4)	
**EBV**			1.000
negative	161 (95.3)	312 (95.1)	
positive	8 (4.7)	16 (4.9)	
**Microsatellite status**			0.297
MSS	153 (90.5)	285 (86.9)	
MSI-H	16 (9.5)	43 (13.1)	
**Depth of invasion**			<0.001
EGC	138 (81.7)	147 (44.8)	
AGC	31 (18.3)	181 (55.2)	
**pT classification**			<0.001
pT1	138 (81.6)	147 (44.8)	
pT2	12 (7.1)	40 (12.2)	
pT3	16 (9.5)	88 (26.8)	
pT4	3 (1.8)	53 (16.2)	
**AJCC stage**			<0.001
IA	143 (84.6)	159 (48.5)	
IB	11 (6.5)	30 (9.1)	
II	8 (4.7)	45 (13.7)	
III	7 (4.2)	94 (28.7)	
**LN metastasis**			<0.001
absent	104 (88.1)	128 (52.5)	
present	14 (11.9)	116 (47.5)	
**Distant metastasis**			0.001
absent	166 (98.2)	293 (89.3)	
present	3 (1.8)	35 (10.7)	
**Local recurrence**			<0.001
absent	169 (100.0)	301 (91.8)	
present	0 (0.0)	27 (8.2)	

Note: BMI, body mass index; WHO, world health organization; LN, lymph node; EBV, Epstein-Barr virus; MSS, microsatellite stable; MSI-H, microsatellite instability high; AJCC, American joint committee on cancer; EGC, early gastric cancer; AGC, advanced gastric cancer.

### 3.2 Elevated PPIB Expression Serves as an Independent Prognostic Indicator for Reduced Survival in Gastric Cancer

**Figure 2:**
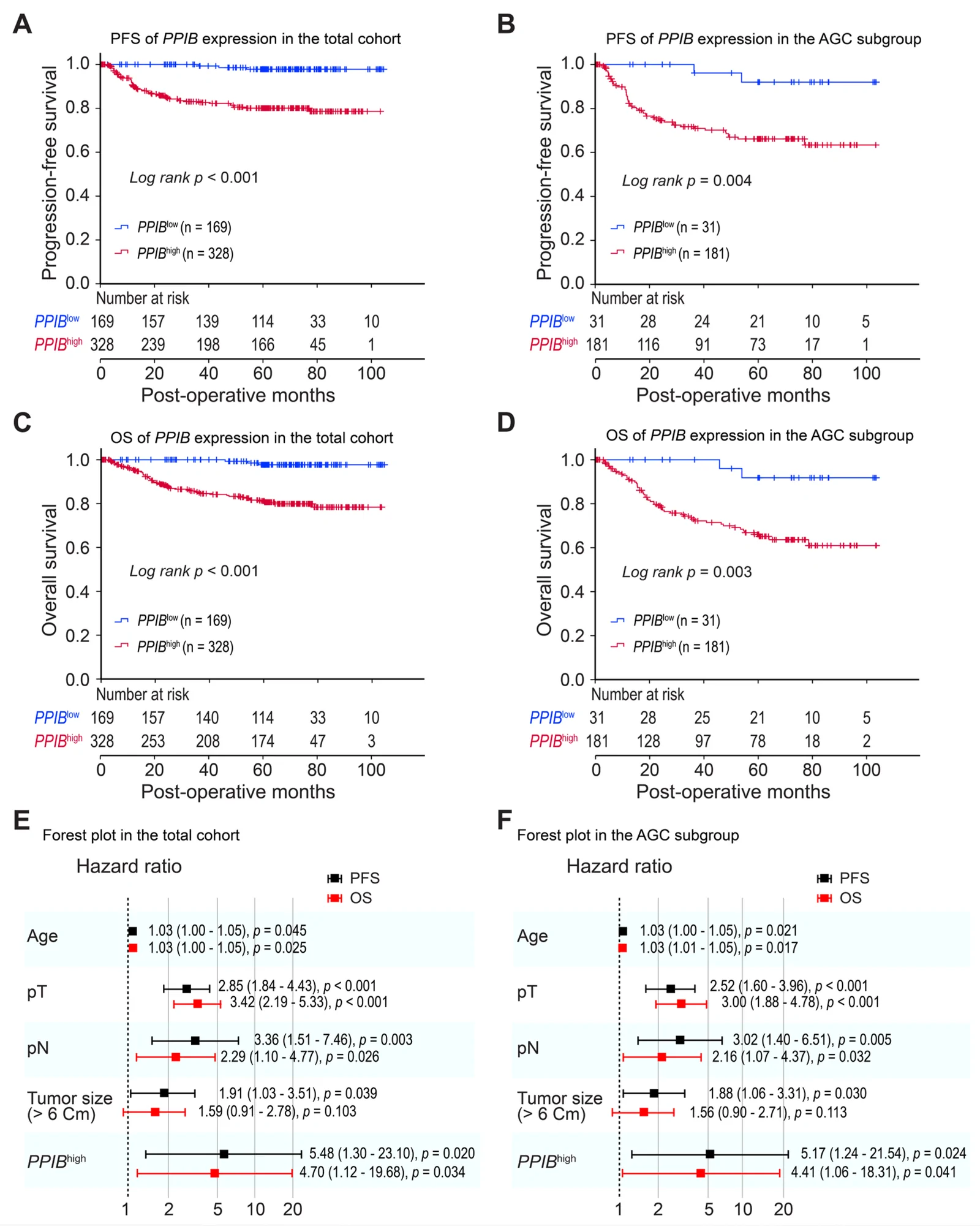
Prognostic significance of *PPIB* expression in gastric cancer. (**A**) Kaplan-Meier survival curves for progression-free survival (PFS) in the total cohort and (**B**) in the AGC subgroup according to *PPIB* expression. (**C**) Overall survival (OS) curves for the total cohort and (**D**) for the AGC subgroup stratified by *PPIB* expression levels. (**E**) Forest plots of multivariate Cox regression analyses for PFS and OS in the total cohort; (**F**) multivariate Cox regression analysis of the AGC subgroup, demonstrating *PPIB* as an independent prognostic factor. Tick marks indicate censored observations, and *p* values from log-rank test are indicated in each panel.

Survival data revealed that advancing pathological pT was a strong indicator of treatment failure; specifically, patients with pT3 and pT4 disease exhibited markedly lower 5-year PFS (73.3% and 40.1%) and the 5-year OS (75.9% and 32.1%) compared to those with earlier-stage tumors (both *p* < 0.001; Supplementary Fig. S3A,B). High *PPIB* (*PPIB*^high^) expression was robustly associated with unfavorable survival (Fig. 2). The PFS rate in the *PPIB*^high^ group was significantly lower than that of the *PPIB* low (*PPIB*^low^) expression cohort (82.6% vs. 98.2%; log-rank *p* < 0.001; Fig. 2A), and this prognostic impact was most pronounced in the AGC subgroup (5-year PFS: 70.7% vs. 93.5%; log-rank *p* = 0.004; Fig. 2B). A similar trend was observed for OS, which was significantly reduced in patients with *PPIB*^high^ expression (83.2% vs. 98.2%; log-rank *p* < 0.001; Fig. 2C) and further amplified in the AGC subgroup (5-year OS: 69.6% vs. 93.5%; log-rank *p* = 0.003; Fig. 2D). Notably, the prognostic impact of *PPIB* expression differed according to tumor stage, as PPIB expression did not significantly stratify survival outcomes in the EGC subgroup (Supplementary Fig. S3C,D). While the absolute difference in PFS and OS between *PPIB*^high^ and *PPIB*^low^ groups was 15.6% and 15.0% in the total cohort, these discrepancies were markedly amplified to 22.8% and 23.9% in the AGC subgroup, respectively. These findings suggest that PPIB serves as a highly sensitive prognostic determinant, particularly for patients with a high tumor burden.

### 3.3 PPIB Expression Independently Predicts Survival Outcomes and Complements Traditional TNM Staging

Cox proportional hazards models were employed to identify independent prognostic factors. In the univariate analysis of the total cohort, *PPIB*^high^ status emerged as a significant predictor of both inferior PFS (HR = 11.81; 95% CI, 3.70–37.72; *p* < 0.001) and OS (HR = 11.33; 95% CI, 3.54–36.22; *p* < 0.001; Table 2). After adjusting for potential confounders in the multivariate model, high *PPIB* expression remained a significant independent predictor for both PFS (HR = 5.48; 95% CI, 1.30–23.10; *p* = 0.020) and OS (HR = 4.70; 95% CI, 1.12–19.68; *p* = 0.034). Pathological T and N stages also maintained their independent prognostic value (Table 2). Furthermore, the robust independent prognostic value of PPIB was consistently observed within the AGC subgroup analysis. As shown in the forest plots, high *PPIB* expression independently predicted shortened PFS (HR = 5.17; 95% CI, 1.24–21.54; *p* = 0.024; Fig. 2E) and OS (HR = 4.41; 95% CI, 1.06–18.31; *p* = 0.041; Fig. 2F; Supplementary Table S2). The predictive performance of the conventional TNM staging system alone was compared with a combined model (TNM + PPIB) to determine whether PPIB provides incremental prognostic information. The integration of PPIB significantly improved the model’s prognostic accuracy. Specifically, the AUC for recurrence increased from 0.846 in the TNM only model to 0.860 in the combined model (*p* = 0.006). Similarly, Harrell’s C-index for OS showed a statistically significant improvement from 0.891 to 0.895 (*p* = 0.022). These *p* values were calculated using a bootstrapping method with 500 iterations (Supplementary Table S3). Collectively, these data suggest that PPIB expression provides critical prognostic utility that complements traditional TNM staging in identifying gastric cancer patients at the highest risk for recurrence and mortality.

**Table 2:** Univariate and multivariate analyses of PFS and OS according to clinicopathologic variables and *PPIB* expression in gastric cancer.

Risk Factor	PFS Hazard Ratio [95% CI]		OS Hazard Ratio [95% CI]	
	Univariate, *p* Value	Multivariate, *p* Value	Univariate, *p* Value	Multivariate, *p* Value
**Age**	1.02 [1.00–1.05], 0.038	1.03 [1.00–1.05], 0.045	1.03 [1.00–1.05], 0.029	1.03 [1.00–1.05], 0.025
**Gender**	1.15 [0.67–1.99], 0.606	NA	1.20 [0.69–2.08], 0.511	NA
**Lauren class**	1.50 [1.10–2.05], 0.010	0.85 [0.60–1.20], 0.343	1.58 [1.16–2.17], 0.004	0.88 [0.63–1.23], 0.444
**pT**	3.76 [2.83–4.99], <0.001	2.85 [1.84–4.43], <0.001	5.05 [3.59–7.12], <0.001	3.42 [2.19–5.33], <0.001
**pN**	14.47 [6.83–30.67], <0.001	3.36 [1.51–7.46], 0.003	11.23 [5.67–22.24], <0.001	2.29 [1.10–4.77], 0.026
**BMI**	0.52 [0.33–0.82], 0.004	0.85 [0.56–1.28], 0.429	0.45 [0.28–0.71], <0.001	0.82 [0.56–1.21], 0.315
**MSI**	1.96 [1.02–3.78], 0.043	1.10 [0.52–2.34], 0.811	1.37 [0.65–2.90], 0.403	NA
**Tumor size**	6.97 [4.18–11.63], <0.001	1.91 [1.03–3.51], 0.039	7.25 [4.30–12.24], <0.001	1.59 [0.91–2.78], 0.103
***PPIB* ^high^**	11.81 [3.70–37.72], <0.001	5.48 [1.30–23.10], 0.020	11.33 [3.54–36.22], <0.001	4.70 [1.12–19.68], 0.034

Note: PFS, progression-free survival; OS, overall survival; CI, confidence interval; NA, not applicable; PPIB, peptidyl-prolyl isomerase B.

### 3.4 PPIB Promotes Oncogenic Phenotypes in Gastric Cancer Cells

**Figure 3:**
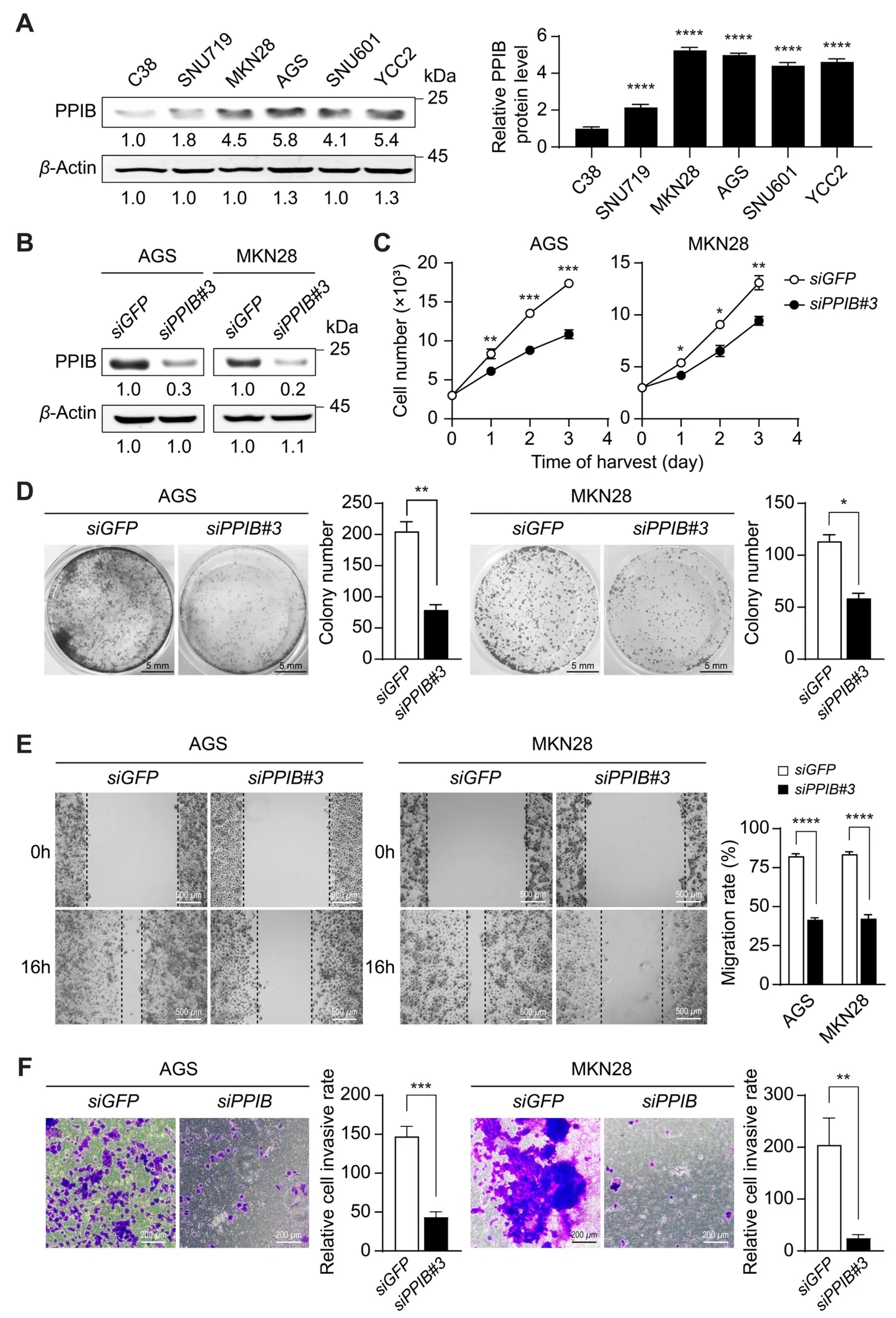
Silencing of PPIB suppresses the proliferation, colony formation, migration, and invasion of gastric cancer cells. (**A**) Protein levels of PPIB in human bronchial epithelial (HBE) cells (C38) and gastric cancer cells (SNU719, MKN28, AGS, SNU601, and YCC2) were determined by Western blot. *β*-Actin was included as an internal loading control. Numbers below blot images indicate the expression as measured by the normalized relative expression level. Graph depicts the experimental quantification based on at least three independent experiments. (**B**) PPIB protein levels in these cells were determined by Western blot. *β*-Actin was included as an internal loading control. Numbers below blot images indicate the expression as measured by the normalized relative expression level. (**C**) Proliferation rate of AGS and MKN28 cells following PPIB silencing, determined by Trypan blue staining at indicated time points. (**D**) Colony formation assay showing the long-term growth inhibitory effects of *PPIB* knockdown. Scale bars = 5 mm. (**E**) Wound healing assays representative of the migratory capacity of AGS and MKN28 cells transfected with *siGFP* or *siPPIB*. Photographs were captured at 0 and 16 h after scratching the confluent cell monolayer. Scale bars = 500 μm. The bar graph shows the calculated migration rate (%). (**F**) Transwell invasion assay evaluating the invasive potential of AGS and MKN28 cells after PPIB knockdown. Representative light micrographs (left) show invaded cells stained with crystal violet. Scale bars = 200 μm. The bar graphs (right) represent the relative cell invasive rate. Data represent the mean ± SD from triplicate experiments. *p < 0.05, **p < 0.01, ***p < 0.001, and ****p < 0.0001 by Student’s *t*-test (two-tailed, unpaired).

We first profiled PPIB protein levels in several human gastric cancer cell lines (SNU719, MKN28, AGS, SNU601 and YCC2) and non-tumorigenic HBE C38 cells. We found that the level of PPIB protein was significantly higher in gastric cancer cells compared with normal cells (Fig. 3A), suggesting that PPIB could be involved in cancer progression. To verify elucidate the specific oncogenic roles of PPIB while strictly ruling out potential siRNA-induced off-target artifacts, we initially engineered and evaluated three independent siRNA candidates (*siPPIB*#1, #2, and #3) targeting distinct nucleotide coordinates of the *PPIB* transcript (Supplementary Fig. S4A). Multi-sequence knockdown validation in MKN28 cells demonstrated that these independent candidates efficiently suppressed PPIB protein levels (Supplementary Fig. S4B) and consistently reproduced a robust, concomitant reduction in both cell proliferation kinetics (Supplementary Fig. S4C) and invasive capacities (Supplementary Fig. S4D). Having firmly established the sequence-specific phenotypic consequences of PPIB silencing, we selected the most potent *siPPIB*#3 sequence to robustly knock down PPIB expression in both AGS and MKN28 cells for comprehensive downstream phenotypic assays (Fig. 3B).

Compared to their corresponding siGFP-transfected counterparts, *siPPIB*-transfected AGS and MKN28 cells exhibited a drastic impediment in longitudinal proliferation rates over a 3-day period (Fig. 3C). Furthermore, clonogenic assays demonstrated that *PPIB* silencing severely compromised both the number and size of colonies, corroborating its requirement for long-term cell survival and proliferative potential (Fig. 3D). Beyond cell growth, scratch-wound healing assays demonstrated that cellular motility was profoundly suppressed 16 h post-scratching in *siPPIB*-transfected cells compared to control cells (Fig. 3E). To explicitly evaluate whether this reduced motility translates to diminished invasive capabilities through the extracellular matrix, we performed Transwell invasion assays, which revealed a substantial reduction in the invasive capacity of both AGS and MKN28 cells upon *PPIB* knockdown (Fig. 3F).

Given that the STAT3 signaling cascade is a well-established driver of oncogenesis, cell survival, and invasion in gastric cancer, we next sought to investigate whether PPIB exerts its oncogenic functions via this pathway. Notably, downstream mechanistic analysis by Western blot showed that silencing of *PPIB* led to a near-complete ablation of STAT3 phosphorylation at its active site in both AGS and MKN28 cells (Supplementary Fig. S5). Crucially, this regulatory effect was highly selective, as total STAT3 protein expression levels remained entirely unaltered regardless of PPIB status (Supplementary Fig. S5). Taken together, these results collectively demonstrate that PPIB acts as a pivotal oncogenic driver in human gastric cancer by accelerating cell proliferation, clonogenicity, motility, and invasive potential, a process tightly coupled with the selective activation of the STAT3 signaling axis.

### 3.5 PPIB Enhances the Oncogenic Potential of Human Gastric Cancer and HBE Cells via STAT3 Activation

**Figure 4:**
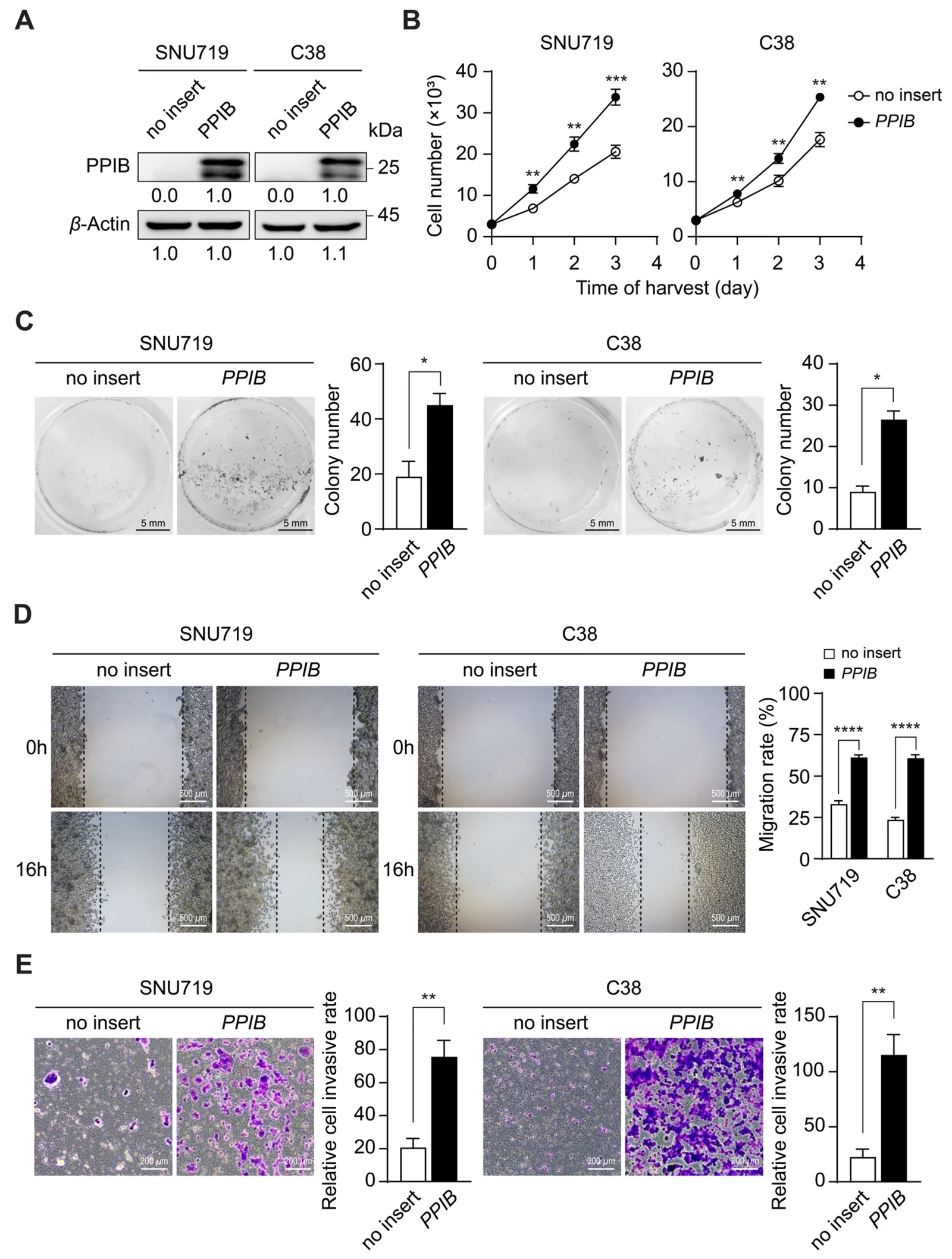
PPIB promotes proliferation, colony formation, migration, and invasion of gastric cancer and HBE cells. (**A**) PPIB protein levels in these cells were determined by Western blot. *β*-Actin was included as an internal loading control. Numbers below blot images indicate the expression as measured by the normalized relative expression level. (**B**) Proliferation rate of C38 and SNU719 cells following PPIB overexpression, assessed by trypan blue exclusion assay at indicated time points. (**C**) Colony formation assay illustrating the enhanced long-term growth potential of cells overexpressing PPIB. 500 cells were plated in 12-well plates and cultured for 1 week and formed colonies were stained with crystal violet. (**D**) Wound healing assays assessing the baseline migratory capacity of SNU719 and C38 cells at 0 and 16 h post-scratching (left; scale bars = 500 μm), with the corresponding bar graph plotting the percentage migration rate (right). (**E**) Transwell invasion assays showing representative light micrographs of crystal violet-stained cells that successfully penetrated the artificial matrix barrier (left; scale bars = 200 μm) and the calculated relative cell invasive rates (right). Data represent the mean ± SD from triplicate experiments. *p < 0.05, **p < 0.01, ***p < 0.001, and ****p < 0.0001 by unpaired, two-tailed Student’s *t*-test.

Given that PPIB depletion significantly attenuated the malignant features of gastric cancer cells, we reasoned that ectopic overexpression of PPIB in cells with lower baseline expression could conversely drive these oncogenic properties. To test this, we stably overexpressed FLAG-tagged PPIB in SNU719 gastric cancer cells and non-tumorigenic C38 epithelial cells, both of which exhibit relatively lower endogenous PPIB levels (Fig. 4A). Indeed, ectopic expression of PPIB significantly accelerated the longitudinal proliferation kinetics of both SNU719 and C38 cells over a 3-day culture period compared to their respective controls (Fig. 4B). Consistently, clonogenic assays revealed that PPIB overactivation substantially enhanced long-term survival and clonogenic efficiency, resulting in a marked increase in colony number (Fig. 4C). We next assessed whether elevated PPIB levels could exacerbate aggressive cellular motility and invasion. Confluent scratch-wound healing assays demonstrated a profound acceleration in horizontal migration rates in PPIB-overexpressing cohorts within 16 h (Fig. 4D). Furthermore, Transwell invasion assays confirmed that ectopic PPIB expression drastically boosted the vertical invasive capacity of both cell lines through the extracellular matrix layer (Fig. 4E), collectively demonstrating that PPIB expression plays a critical role in promoting both proliferative expansion and aggressive motility in both malignant and non-tumorigenic epithelial contexts.

**Figure 5:**
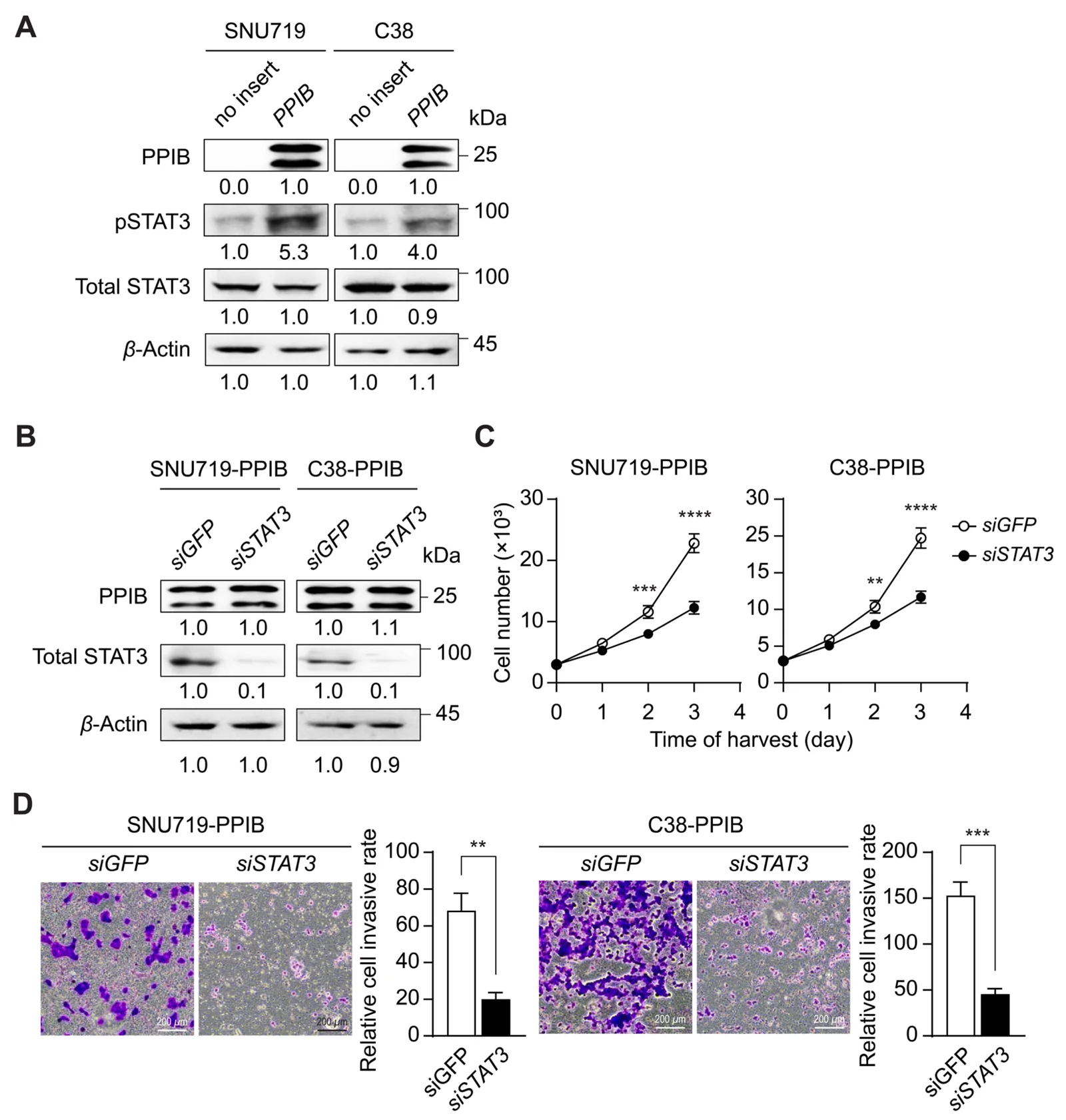
PPIB driven proliferation and invasion are dependent on the STAT3 signaling axis. (**A**) Western blot analysis of PPIB, phosphorylated STAT3 (pSTAT3), and total STAT3 levels in SNU719 and C38 cells stably overexpressing PPIB (PPIB) or empty vector controls (no insert), with *β*-Actin as a loading control and densitometric normalized relative expression levels shown below. (**B**) Western blot validation of STAT3 silencing efficiency in stable PPIB-overexpressing lines (SNU719-PPIB and C38-PPIB) transfected with control siRNA (*siGFP*) or STAT3-targeted siRNA (*siSTAT3*). (**C**) Cell proliferation kinetics tracking the growth rates of SNU719-PPIB and C38-PPIB cells over a 3-day harvest timeline following transfection with *siGFP* or *siSTAT3*. (**D**) Representative light micrographs (scale bars = 200 μm) and corresponding quantification of Transwell invasion assays showing that STAT3 knockdown rescues the hyper-invasive phenotype induced by PPIB overactivation. Data represent the mean ± SD from triplicate experiments; **p < 0.01, ***p < 0.001, and ****p < 0.0001 versus *siGFP* by two-tailed, unpaired Student’s *t*-test.

To delineate the molecular mechanism underpinning these PPIB-induced phenotypic enhancements, we investigated the downstream activation status of the STAT3 signaling pathway. Western blot analysis revealed that stable overexpression of PPIB led to a upregulation of pSTAT3 at its transactivation domain in both SNU719 and C38 cells, while total STAT3 protein expression levels remained completely unchanged (Fig. 5A). To establish a definitive functional link between this selective STAT3 activation and the observed PPIB-driven phenotypes, we performed a genetic rescue experiment by silencing endogenous STAT3 in the PPIB-overexpressing cells using specific *siSTAT3* (Fig. 5B). Importantly, the hyperproliferative phenotypes triggered by PPIB overactivation were significantly blunted upon STAT3 depletion in both SNU719-PPIB and C38-PPIB lines (Fig. 5C). Similarly, the accelerated cell invasion induced by PPIB overexpression was completely rescued and abrogated back to baseline levels following *siSTAT3* transfection (Fig. 5D). Taken together, these gain-of-function and epistasis data demonstrate that PPIB acts as a potent upstream positive regulator of the STAT3 signaling cascade, and that its capacity to promote cellular growth, clonogenicity, and invasive progression is strictly dependent on the activation of the STAT3 signaling axis.

## 4 Discussion

Reliable biomarker identification for risk stratification in gastric cancer remains a clinical priority, particularly for patients in advanced stages where prognosis is highly variable. In this study, we demonstrated that *PPIB* is a robust and independent prognostic factor for both PFS and OS in gastric cancer. Notably, our subgroup analysis revealed that the prognostic power of *PPIB* is even more pronounced in AGC and provides critical insights into the management of high-risk patients including those with node-negative disease. In contrast, the exceptionally high survival rate observed in the *PPIB*^low^ group can be attributed to its clinicopathological composition, where 84.6% (143/169) of patients presented with Stage IA disease. The identical metrics for PFS and OS in this group further reflect the favorable prognosis of early-stage disease, as no recurrence events (0/169) were recorded during the follow-up period.

Accurate quantification of biomarkers is essential for robust risk stratification and the implementation of precision oncology, particularly in identifying patients most likely to benefit from targeted therapies. Previous assessments of PPIB expression in malignancy have largely relied on immunohistochemistry, a protein-based approach often limited by poor reproducibility. This limitation reflects both antibody variability across batches and the subjectivity inherent to semi-quantitative scoring methods [29,30,31,32]. To overcome these challenges, we employed RNAscope ISH, a highly sensitive and specific platform that utilizes a unique double Z-probe design to minimize background signal [33,34,35]. Building on our prior validation in pulmonary adenocarcinoma [19], we confirmed that transcript-level detection effectively identified low-abundance markers that may be missed by protein-based assays. Although clinical quantification was performed at the transcript level using RNAscope ISH, our Western blot analysis (Fig. 3A) across multiple cell lines confirmed that PPIB protein levels consistently reflect the observed mRNA trends, supporting the functional relevance of our findings.

The association between PPIB expression and adverse clinicopathological features aligns with the emerging role of cyclophilins in tumor progression. PPIB is known to function as a molecular chaperone that facilitates the folding of type I collagen and has been implicated in the activation of signaling pathways that drive epithelial-mesenchymal transition [17,23,36]. We found that elevated PPIB expression relates to deeper wall invasion and increased tumor size, which suggests that this protein functions as a primary driver of local tumor aggressiveness. Functional assays provide mechanistic support for these clinical observations, demonstrating that siRNA-mediated depletion of PPIB severely compromises the proliferation, clonogenicity, motility, and invasion of gastric cancer cells, whereas its ectopic overexpression in PPIB-low malignant (SNU719) and non-tumorigenic epithelial (C38) cells is sufficient to induce these aggressive oncogenic phenotypes. These results suggest that PPIB expression may contribute significantly to the oncogenic potential of gastric cells, positioning it as a candidate for future therapeutic intervention studies. Based on prior literature, this oncogenic activity was hypothesized to be sustained through a regulatory circuit involving the STAT3 signaling pathway; for instance, inflammatory signals like IL-6 can induce the translocation of PPIB into the nucleus to interact with STAT3, and the loss of miR-520d-5p can create a feedback loop that maintains constant STAT3 activation [23]. Aligning with this prior study, our loss-of-function data demonstrate that *PPIB* silencing leads to a profound and selective ablation of pSTAT3 at its active site without altering total STAT3 protein levels. Furthermore, our genetic epistasis rescue experiments firmly establish a definitive causal link, as the hyperproliferation and hyper-invasiveness triggered by ectopic PPIB overexpression were systematically blunted and rescued back to baseline levels upon concurrent STAT3 knockdown via specific *siSTAT3*. Consequently, this rigorous validation positions PPIB not merely as a passive prognostic biomarker, but as a central upstream signaling modulator of the STAT3 cascade, rendering it a highly compelling and attractive candidate for future targeted therapeutic interventions in gastric cancer [37,38].

Pathological node-negative (pN0) status is traditionally heralded as a hallmark of favorable prognosis; however, the non-negligible rate of early relapse in this cohort highlights the limitations of anatomical staging in capturing the occult biological aggressiveness of AGC [39,40,41,42]. In the present study, the prognostic impact of PPIB, which was statistically significant in the overall AGC cohort (PFS, *p* = 0.004; OS, *p* = 0.003), was consistently observed across nodal subgroups. Notably, in the pN0 subgroup (n = 83), no events were observed in patients with PPIB-low expression during follow-up, whereas PPIB-high expression was associated with a trend toward poorer survival (PFS, *p* = 0.103; OS, *p* = 0.062). A similar trend was observed in pN+ patients (PFS, *p* = 0.057; OS, *p* = 0.082; Supplementary Fig. S6). These findings suggest that PPIB may serve as an independent marker of aggressive tumor biology, rather than merely reflecting nodal status. The quantitative improvement in AUC and C-index values confirms that *PPIB* expression adds significant incremental value to the traditional TNM staging system. This suggests that *PPIB* serves as a molecular indicator of occult biological aggressiveness, enabling more refined risk stratification, particularly in advanced stages where clinical outcomes are highly variable. The comparable survival patterns observed in both pN0 and pN+ groups further support the notion that the oncogenic role of PPIB may extend beyond conventional lymphatic dissemination, potentially reflecting intrinsic tumor aggressiveness associated with dysregulated intracellular proteostasis [43]. Our functional assays and genetic rescue data provide clear experimental validation for these clinical observations, proving that PPIB drives proliferation and invasion in a STAT3 dependent manner. Mechanistically, PPIB has been reported to activate STAT3 signaling, a key regulator of VEGF expression, suggesting a potential link to tumor angiogenesis [15,44,45]. In pN0 patients, where lymphatic spread is absent, this enhanced tumor vascularity and active PPIB-STAT3 axis may facilitate hematogenous dissemination, providing a compelling molecular rationale for the early recurrence observed in this high-risk cohort [46,47,48]. Consistently, multivariate analysis confirmed that PPIB retains independent prognostic significance in the overall AGC cohort (OS: HR = 4.41, *p* = 0.041), even after adjustment for age, pT, and pN stage. Although the limited number of patients with low PPIB expression in both subgroups may have reduced statistical power, the consistent trend observed across analyses support further evaluation of PPIB as a biomarker for identifying high-risk patients, even within anatomically early-stage disease. Future large-scale, multicenter prospective studies, together with mechanistic investigations, are warranted to validate the prognostic utility of PPIB and to further elucidate its biological role in tumor progression, angiogenesis, and metastasis in advanced gastric cancer.

Despite the meaningful findings of this study, several limitations should be considered when interpreting the results. First, this investigation was based exclusively on specimens collected from a single institution, which may introduce institutional selection bias and limit the generalizability of the findings to other populations. Furthermore, the retrospective nature of this single center study and the use of a data driven threshold for PPIB expression may introduce inherent risks of bias and overfitting. While we attempted to mitigate these concerns through internal validation via bootstrapping and sensitivity analyses, the absence of an independent external validation cohort requires that our findings be confirmed in prospective multicenter settings. Second, the present study employed the RNAscope assay, which is a relatively costly and technically specialized platform, potentially limiting the practical reproducibility and broad clinical implementation of this approach in routine diagnostic settings. Although 2 mm TMA cores were selected to ensure representativeness, single section analysis may not capture the full spatial complexity of the tumor, and the reliance on specialized automated systems may limit immediate integration into routine diagnostic workflows. Third, although a substantial number of AGC patients were included, complete data regarding treatment related variables including granular information on adjuvant treatment modalities, the number of surgical procedures, the receipt of chemotherapy, and exact treatment responses were not consistently available. Consequently, the possible confounding effects of these therapeutic factors on the observed survival and clinicopathological results could not be fully evaluated. Therefore, further prospective multicenter studies involving larger patient cohorts and comprehensive clinical data are warranted to validate our findings and to better define their clinical applicability. Nevertheless, the present study provides a significant clinical observation of PPIB prognostic relevance in advanced gastric cancer, establishing a robust scientific foundation that underscores its practical utility in clinical oncology.

## 5 Conclusion

In summary, PPIB emerges as an independent prognostic indicator linked to adverse clinical outcomes in advanced gastric cancer, with our functional evidence suggesting that it contributes to aggressive tumor proliferation and invasion at least in part through downstream STAT3 activation. These findings suggest that incorporating PPIB into prognostic models may enhance risk assessment beyond conventional TNM staging, supporting more individualized therapeutic approaches. Furthermore, by elucidating the link between PPIB and STAT3 signaling, this study establishes a foundation for evaluating the PPIB-STAT3 axis as a potential therapeutic target in advanced gastric cancer.

## Data Availability

The datasets generated and analyzed during the current study are not publicly available due to institutional restrictions and patient confidentiality protocols. However, the data are available from the Corresponding Authors, [Seoung Wan Chae and Joon-Yong Chung], upon reasonable request and with permission from the IRB of the Kangbuk Samsung Hospital.

## Supplementary Materials

The supplementary material is available online at https://www.techscience.com/doi/10.32604/or.2026.083437/s1.

## Abbreviations

AGC	Advanced Gastric Cancer
AJCC	American Joint Committee on Cancer
AUC	Area Under the Curve
BMI	Body Mass Index
CI	Confidence Interval
C-index	Concordance Index
CypB	Cyclophilin B
DAB	3,3′-Diaminobenzidine
EBER-ISH	Epstein–Barr Virus-Encoded RNA *In Situ* Hybridization
EBV	Epstein–Barr Virus
EGC	Early Gastric Cancer
ER	Endoplasmic Reticulum
FFPE	Formalin-Fixed, Paraffin-Embedded
H&E	Hematoxylin and Eosin
HBE	Human Bronchial Epithelial
HR	Hazard Ratio
ICC	Intraclass Correlation Coefficient
ISH	*In Situ* Hybridization
LVI	Lymphovascular Invasion
MMR	Mismatch Repair
MSI	Microsatellite Instability
MSI-H	Microsatellite Instability-High
MSI-L	Microsatellite Instability-Low
MSS	Microsatellite Stable
OS	Overall Survival
PFS	Progression-Free Survival
PNI	Perineural Invasion
pN	Pathological Nodal Stage
PPIB	Peptidyl-Prolyl Isomerase B
pT	Pathological Tumor Stage
RNAscope	RNA *In Situ* Hybridization Technology
siRNA	Small Interfering RNA
TMA	Tissue Microarray
TNM	Tumor Node Metastasis
WHO	World Health Organization
