# Supplementary material for: Prognostic Significance and Functional Role of PPIB in a Retrospective Cohort of Patients with Advanced Gastric Cancer
Source: Oncol Res. 2026 Aug 13;34(9):22. doi: 10.32604/or.2026.083437 (PMC13494475; doi:10.32604/or.2026.083437)
Supplement: Supplementary file 1 [file OncolRes-34-83437-s001.zip › TSP_OR_83437-s001.docx]

| **Supplementary Table S1:** Clinicopathologic characteristics of patients with gastric cancer | |  |
| --- | --- | --- |
| Category (n = 497) | n (%) | |
| Age (years) |  | |
| ≤ 60 | 218 (43.9) | |
| > 60 | 279 (56.1) | |
| Tumor size (cm) |  | |
| ≤ 6 | 401 (80.7) | |
| > 6 | 96 (19.3) | |
| Gender |  | |
| male | 357 (71.8) | |
| female | 140 (28.2) | |
| BMI |  | |
| underweight (< 18.5) | 32 (6.4) | |
| normal (18.5-24.9) | 310 (62.4) | |
| overweight (25.0-29.9) | 135 (27.2) | |
| obesity (≥ 30) | 19 (3.8) | |
| missing | 1 (0.2) | |
| Signet-ring cell component |  | |
| absent | 341 (68.8) | |
| present | 156 (31.4) | |
| WHO classification |  | |
| tubular | 289 (58.2) | |
| poorly cohesive | 121 (24.3) | |
| mucinous | 5 (1.0) | |
| carcinoma with lymphoid stroma | 17 (3.4) | |
| mixed | 65 (13.1) | |
| Lauren classification |  | |
| intestinal | 288 (57.9) | |
| diffuse | 129 (26.0) | |
| mixed | 80 (16.1) | |
| Resection margin |  | |
| absent | 489 (98.4) | |
| present | 8 (1.6) | |
| Lymphatic invasion |  | |
| absent | 345 (69.4) | |
| present | 152 (30.6) | |
| Vascular invasion |  | |
| absent | 457 (92.0) | |
| present | 40 (8.0) | |
| Lymphovascular invasion |  | |
| absent | 337 (67.8) | |
| present | 160 (32.2) | |
| Perinueral invasion |  | |
| absent | 389 (78.3) | |
| present | 108 (21.7) | |
| EBV |  | |
| negative | 473 (95.2) | |
| positive | 24 (4.8) | |
| Microsatellite status (MSS) |  | |
| MSS | 438 (88.1) | |
| MSI-H | 59 (11.9) | |
| Depth of invasion |  | |
| EGC | 285 (57.3) | |
| AGC | 212 (42.7) | |
| pT classification |  | |
| pT1 | 285 (57.3) | |
| pT2 | 52 (10.5) | |
| pT3 | 104 (20.9) | |
| pT4 | 56 (11.3) | |
| AJCC stage |  | |
| IA | 302 (60.8) | |
| IB | 41 (8.2) | |
| II | 53 (10.7) | |
| III | 101 (20.3) | |
| LN metastasis |  | |
| absent | 232 (46.7) | |
| present | 130 (26.1) | |
| not assessed | 135 (27.2) | |
| Distant metastasis |  | |
| absent | 459 (92.4) | |
| present | 38 (7.6) | |
| *PPIB* |  | |
| low | 169 (34.0) | |
| high | 328 (66.0) | |
| Local recurrence |  | |
| absent | 407 (94.9) | |
| present | 22 (5.1) | |

Note: BMI, body mass index; WHO, world health organization; EBV, Epstein-Barr virus; MSS, microsatellite stable; MSI-H, microsatellite instability high; EGC, early gastric cancer; AGC, advanced gastric cancer; LN, lymph node; American Joint Committee on Cancer; PPIB, peptidylprolyl isomerase B. LN metastasis was evaluated only in patients who underwent LN dissection (n = 362).

**Supplementary Table S2:** Univariate and multivariate analyses of PFS and OS according to clinicopathologic variables and *PPIB* expression in the AGC subgroup

| Risk factor | PFS Hazard ratio [95%CI] | | | |  | OS Hazard ratio [95%CI] | | | |
| --- | --- | --- | --- | --- | --- | --- | --- | --- | --- |
|  | Univariate | *p* value | Multivariate | *p* value |  | Univariate | *p* value | Multivariate | *p* value |
| Age | 1.02 [1.00 - 1.05] | 0.029 | 1.03 [1.00 - 1.05] | 0.021 |  | 1.03 [1.00 - 1.05] | 0.014 | 1.03 [1.01 - 1.05] | 0.017 |
| Gender | 1.10 [0.62 - 1.92] | 0.751 | NA |  |  | 1.11 [0.64 – 1.92] | 0.719 | NA |  |
| Lauren classification | 0.95 [0.69 - 1.32] | 0.764 | NA |  |  | 0.98 [0.71 – 1.34] | 0.878 | NA |  |
| pT classification | 3.726 [2.41 - 5.75] | < 0.001 | 2.52 [1.60 – 3.96] | < 0.001 |  | 4.15 [2.67 – 6.44] | < 0.001 | 3.00 [1.88 – 4.78] | < 0.001 |
| pN classification | 5.10 [2.41 - 10.81] | < 0.001 | 3.02 [1.40 – 6.51] | 0.005 |  | 3.93 [1.98 – 7.78] | < 0.001 | 2.16 [1.07 - 4.37] | 0.032 |
| BMI | 0.96 [0.89 - 1.05] | 0.369 | NA |  |  | 0.92 [0.85 - 1.00] | 0.060 | 0.97 [0.90 – 1.04] | 0.385 |
| MSI | 1.55 [0.78 - 3.07] | 0.213 | NA |  |  | 1.05 [0.50 - 2.22] | 0.900 | NA |  |
| Tumor size (> 6 cm) | 3.30 [1.91 - 5.69] | < 0.001 | 1.88 [1.06 - 3.31] | 0.030 |  | 2.85 [1.68 – 4.84] | < 0.001 | 1.56 [0.90 - 2.71] | 0.113 |
| *PPIB*^high^ | 6.19 [1.51 - 25.41] | 0.011 | 5.17 [1.24 - 21.54] | 0.024 |  | 6.29 [1.53 – 25.81] | 0.011 | 4.41 [1.06 - 18.31] | 0.041 |

Note: PFS, progression-free survival; OS, overall survival; CI, confidence interval; NA, not applicable; BMI, body mass index; MSI, microsatellite instability; PPIB, peptidylprolyl isomerase B.

| **Supplementary Table S3:** Comparison of predictive performance between TNM and TNM+PPIB Models | | | |  |
| --- | --- | --- | --- | --- |
| Metric | TNM stage (95% CI) | TNM stage + PPIB (95% CI) | *p* value | |
| AUC (Recurrence) | 0.846 (0.804-0.896) | 0.860 (0.823-0.904) | 0.006 | |
| C-index (OS) | 0.891 (0.860-0.924) | 0.895 (0.867-0.930) | 0.022 | |

Note: TNM, tumor node metastasis; AUC, area under the curve; C-index, concordance index; CI, confidence interval; PPIB, peptidylprolyl isomerase B.

**Supplementary Fig. S1:** Flowchart of the study population and patient stratification for *PPIB* mRNA analysis.

**Supplementary Fig. S2:** Internal validation of the optimal *PPIB* cut-off value via bootstrapping. The stability of the identified optimal threshold (2.74) was evaluated using a bootstrap distribution analysis with 500 iterations.

S**upplementary Fig. S3:** Survival analyses according to pT stage and *PPIB* expression. Advanced pT stage is associated with shorter PFS (**A**, log-rank *p* < 0.001) and poorer OS (**B**, log-rank *p* < 0.001). In the EGC subgroup, *PPIB* expression is not associated with PFS (**C**, log-rank *p* = 0.166) or OS (**D**, log-rank *p* = 0.325). Tick marks indicate censored observations.

**Supplementary Fig. S4:** Multi-sequence siRNA validation confirms that *PPIB* silencing suppresses the proliferation and invasion of gastric cancer cells. (**A**) Schematic mapping of three independent siRNAs (*siPPIB*#1, #2, and #3) targeting distinct nucleotide coordinates of human *PPIB* mRNA (NM_000942.5), including their specific sense strand and target sequences. (**B**) Western blot analysis of PPIB knockdown efficiency in MKN28 gastric cancer cells transfected with control siRNA (*siGFP*) or individual *siPPIB* sequences, with *β*-Actin utilized as a loading control and densitometric fold-changes indicated below. (**C**) Proliferation kinetics of MKN28 cells over a 3-day harvest timeline following transfection with siGFP or sequence-specific *siPPIB* candidates. (**D**) Representative light micrographs and corresponding quantification of Transwell invasion assays showing crystal violet-stained MKN28 cells that penetrated the extracellular matrix. Data represent the mean ± SD from triplicate experiments; ^***^*p* < 0.001 and ^****^*p* < 0.0001 versus *siGFP* by two-tailed, unpaired Student’s t-test.

**Supplementary Fig. S5:** Knockdown of *PPIB* downregulates STAT3 phosphorylation in gastric cancer cells. Western blot analysis of PPIB, phosphorylated STAT3 (pSTAT3), and total STAT3 protein levels in AGS and MKN28 gastric cancer cells transfected with control siRNA (*siGFP*) or siRNA targeting PPIB (*siPPIB*). *β*-Actin was utilized as an internal loading control to ensure equal protein loading. Densitometric quantification values, representing the relative fold-change normalized to the respective *siGFP* control group, are indicated beneath each protein blot panel.

**Supplementary Fig. S6:** Survival analyses according to *PPIB* expression in the AGC subgroups stratified by LN invasion. (**A**) PFS and (**B**) OS in AGC patients without LN invasion show no significant association with *PPIB* expression (*p* = 0.103 and *p* = 0.062, respectively). (**C**) PFS and (**D**) OS in AGC patients with LN invasion also show no significant correlation with *PPIB* expression (*p* = 0.057 and *p* = 0.082, respectively). Tick marks indicate censored observations.
